# Looking Beyond Blood Pressure: A Case-Based Approach to Primary Aldosteronism

**DOI:** 10.7759/cureus.102946

**Published:** 2026-02-04

**Authors:** Pooja Alipuria, Atush Alipuria

**Affiliations:** 1 Internal Medicine, Yashoda Super Speciality Hospital, Ghaziabad, IND; 2 Pulmonology and Critical Care, Yashoda Super Speciality Hospital, Ghaziabad, IND

**Keywords:** adrenal adenoma, aldosterone renin ratio, hypertension, mineralocorticoid receptor antagonist, primary aldosteronism, proteinuria, renin angiotensin system, secondary hypertension, spironolactone, target organ damage

## Abstract

Primary aldosteronism is an underrecognized cause of secondary hypertension, particularly when blood pressure elevation is not resistant and classic features are subtle. We describe a 63-year-old man with long-standing hypertension who presented with generalized weakness and was found to have persistent mild hypokalemia, proteinuria, and concentric left ventricular hypertrophy that appeared disproportionate to the degree of blood pressure elevation. These findings prompted evaluation for primary aldosteronism. Aldosterone-renin ratio testing, performed after correction of hypokalemia and interpreted in the context of ongoing antihypertensive therapy, demonstrated inappropriately elevated aldosterone with suppressed renin. Imaging revealed a unilateral adrenal lesion consistent with an adrenal adenoma. The patient declined surgical intervention and was managed medically with mineralocorticoid receptor antagonist therapy. This resulted in normalization of serum potassium, improved blood pressure control, resolution of proteinuria, and loss of renin suppression on follow-up. This case emphasizes the importance of maintaining a high index of suspicion for primary aldosteronism even when hypertension is not resistant, particularly when mild to moderate blood pressure elevation is accompanied by spontaneous hypokalemia and evidence of renal or cardiac target-organ involvement. An individualized, physiology-based diagnostic approach may facilitate earlier recognition of excess aldosterone and help prevent progression to resistant hypertension and long-term cardiovascular and renal complications.

## Introduction

Primary aldosteronism (PA) is a common and potentially reversible cause of secondary hypertension. The 2016 Endocrine Society Clinical Practice Guideline recommended screening for PA in selected high-risk populations, including individuals with resistant hypertension, spontaneous hypokalemia, adrenal incidentalomas, or early-onset hypertension [1]. Despite these recommendations, PA remains underrecognized, and many patients are diagnosed only after prolonged hypertension and the development of target-organ damage.

Aldosterone excess is associated with cardiovascular and renal injury that may be disproportionate to the degree of blood pressure elevation. Compared with essential hypertension, patients with PA experience higher rates of cardiovascular events [2] and worse long-term renal outcomes [3], supporting the need for earlier recognition of the condition when biochemical abnormalities coexist with evidence of organ involvement.

The 2025 Endocrine Society Clinical Practice Guideline builds on this concept by recommending screening for PA in all individuals with hypertension, emphasizing earlier detection and pragmatic diagnostic strategies [4]. Here, we report a case of PA diagnosed before the development of resistant hypertension, prompted by persistent mild hypokalemia, proteinuria, and concentric left ventricular hypertrophy.

## Case presentation

Patient information

A 63-year-old man with hypertension since his late 30s presented with complaints of generalized weakness and exertional bilateral lower-limb weakness for approximately one year. He had no known history of diabetes mellitus, chronic kidney disease, coronary artery disease, or cerebrovascular disease. There was no significant family history of hypertension or endocrine disorders. He had been taking amlodipine 5 mg and atenolol 50 mg daily for the past five years and was not on any other regular medications. The patient also had a history of a right macular hole diagnosed approximately 10 months earlier, following visual distortion, for which he was managed by an ophthalmologist.

Clinical examination

On examination, the patient was obese. Standardized office blood pressure was 160/90 mmHg, and pulse rate was 80 beats per minute. General physical, systemic, and neurological examinations were unremarkable. According to the patient, home blood pressure recordings over several months consistently ranged between 150-160 and 90-100 mmHg.

Table 1 shows baseline investigations.

**Table 1:** Baseline laboratory findings

Parameter	Result	Reference Range
Serum sodium	140 mmol/L	136-146 mmol/L
Serum potassium	3.3 mmol/L	3.5-5.2 mmol/L
Repeat Serum potassium	3.0 mmol/L	3.5-5.2 mmol/L
Electrocardiogram	Normal sinus rhythm	-
Serum creatinine	1.01 mg/dL	0.70-1.20 mg/dL
Blood urea	25 mg/dL	16.6-48.5 mg/dL
Estimated glomerular filtration rate	74.6 mL/min/1.73 m²	-
Total bilirubin	0.6 mg/dL	0.2-1.2 mg/dL
Aspartate aminotransferase	24 U/L	5-34 U/L
Alanine aminotransferase	26 U/L	0-41 U/L
Total protein	6.8 g/dL	6.6-8.7 g/dL
Serum albumin	3.7 g/dL	3.5-5.2 g/dL
Glycated hemoglobin	6.30%	4.5-6.3%
Fasting plasma glucose	108 mg/dL	65-110 mg/dL
Total cholesterol	230 mg/dL	<200 mg/dL
Low-density lipoprotein cholesterol	143 mg/dL	<99 mg/dL
Triglycerides	190 mg/dL	0-150 mg/dL
High-density lipoprotein cholesterol	49 mg/dL	>30 mg/dL
Thyroid-stimulating hormone	2.91 µIU/mL	0.39-4.2 µIU/mL
Urine routine microscopy (initial)		
Protein	1+	Nil
Pus cells	Nil	Nil
Red blood cells	Absent	Absent
Urine glucose	Negative	Negative
Urine routine microscopy (repeat)		
Protein	1+	Nil
Pus cells	Nil	Nil
Red blood cells	Absent	Absent
Urine glucose	Negative	Negative

Initial laboratory evaluation revealed persistent mild hypokalemia and proteinuria, prompting further assessment (Table 2).

**Table 2:** Renal evaluation Baseline renal evaluation demonstrating persistent subnephrotic proteinuria, which contributed to suspicion of secondary hypertension.

Parameter	Result	Reference Range
Urine albumin-creatinine ratio	0.07	<1.0
24-hour urine protein	594 mg/24 h	40-150 mg/24 h
24-hour urine volume	2700 mL	-

Transthoracic echocardiography, performed as part of hypertension-related target-organ evaluation, demonstrated concentric left ventricular hypertrophy, no regional wall motion abnormality, a left ventricular ejection fraction of 60%, and grade I diastolic dysfunction.

Clinical reasoning

Although hypokalemia was mild, the combination of long-standing hypertension beginning at a young age, persistent hypokalemia associated with neuromuscular symptoms, proteinuria, and evidence of hypertensive target-organ involvement raised a strong suspicion for primary aldosteronism. The hypokalemia was spontaneous, with no history of diuretic use or gastrointestinal losses to account for the electrolyte abnormality.

Antihypertensive optimization prior to ARR (aldosterone-renin ratio) testing

Given uncontrolled blood pressure and renal involvement, antihypertensive therapy was optimized prior to biochemical testing (Table 3).

**Table 3:** Medication modifications undertaken prior to aldosterone-renin ratio assessment ASCVD: atherosclerotic cardiovascular disease; ARR: aldosterone-renin ratio

Step	Intervention	Rationale
Step 1	Amlodipine stopped; telmisartan 40 mg + cilnidipine 10 mg twice daily started	Blood pressure control and renal protection
Step 1	Atenolol 50 mg switched to metoprolol succinate 25 mg for 2 weeks	Safer beta-blocker taper due to shorter half-life and flexible dosing
Step 1	Rosuvastatin 10 mg nightly initiated	Dyslipidemia with estimated 10-year ASCVD risk >10%
Step 2	Metoprolol succinate reduced to 12.5 mg for 2 weeks	Gradual down-titration
Step 3	Metoprolol succinate given on alternate days for 2 weeks, then stopped	Minimize renin suppression and improve interpretation of the aldosterone-renin ratio
Along with Step 2 & 3	Oral potassium supplementation initiated and titrated	Correction of hypokalemia prior to ARR testing

ARR testing was performed two weeks after complete cessation of beta-blocker therapy, once serum potassium had been corrected.

Biochemical evaluation

The aldosterone-renin ratio was measured after normalization of serum potassium. The patient was ambulatory prior to sampling and sat for 15 minutes before blood collection. He was also advised to avoid dietary sodium restriction during the days preceding screening (Table 4).

**Table 4:** Aldosterone-renin results (assay method used chemiluminescence immunoassay)

Parameter	Result	Reference Range
Serum potassium	4.2 mmol/L	3.5-5.2 mmol/L
Aldosterone	25.7 ng/dL	2.21-35.30 ng/dL
Direct renin	1.67 mIU/L	4.40-46.10 mIU/L
Aldosterone-renin ratio	153.6	<20.6

Imaging and additional testing

**Figure 1:**
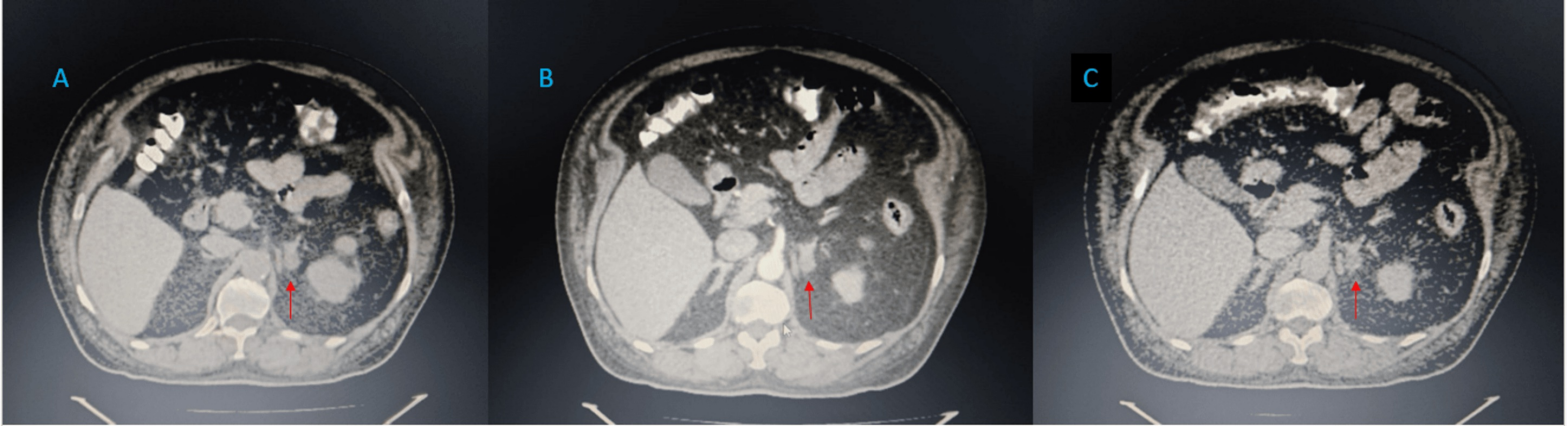
Computed tomography of the adrenal glands demonstrating a left adrenal lesion (A) Non-contrast image showing a small left adrenal lesion with low attenuation (arrow). (B) Arterial phase image demonstrating no significant enhancement (arrow). (C) Venous phase image confirming a non-enhancing lesion (arrow), consistent with a benign adrenal lesion.

Contrast-enhanced adrenal CT demonstrated a left adrenal lobulated, predominantly fat-density lesion measuring 1.3 x 1.1 x 1.2 cm, consistent with a benign adrenal lesion (Figure 1).

A 1-mg overnight dexamethasone suppression test excluded cortisol co-secretion (8 a.m. cortisol 1.1 µg/dL; reference 3.7-19.4 µg/dL). Biochemical testing for pheochromocytoma was not performed because the adrenal lesion demonstrated homogeneous low attenuation on non-contrast CT (-12 Hounsfield units), consistent with a lipid-rich adenoma, and the patient had no clinical features suggestive of catecholamine excess; this approach is supported by current adrenal incidentaloma guidelines [5].

Diagnosis

A diagnosis of primary aldosteronism was made based on the presence of hypertension with spontaneous hypokalemia, a markedly elevated aldosterone-renin ratio with suppressed renin, and identification of a unilateral adrenal lesion on imaging in the absence of lateralization studies. Confirmatory aldosterone suppression testing was not pursued, given the high-probability clinical phenotype [1,4] and anticipated poor adherence to inpatient testing.

Management and follow-up

The patient declined surgical intervention; therefore, adrenal venous sampling was not performed, and medical management was chosen. Spironolactone 25 mg daily was initiated, and telmisartan-cilnidipine was reduced to once daily with close blood pressure monitoring. Oral potassium supplementation was continued for two days after initiating spironolactone and then discontinued. As home blood pressure readings remained elevated (~150/90 mmHg), spironolactone was increased to 50 mg after two weeks.

At four weeks after initiation of spironolactone, repeat evaluation showed (Table 5).

**Table 5:** Biochemical and metabolic findings on follow-up after initiation of mineralocorticoid receptor antagonist therapy

Parameter	Result	Reference Range
Serum potassium	4.8 mmol/L	3.5-5.2 mmol/L
Direct renin	4.69 mIU/L	4.40-46.10 mIU/L
Urine protein	Nil	Nil
Spot urine protein-creatinine ratio	0.18	<0.20
Serum creatinine	1.10 mg/dL	0.70-1.20 mg/dL
Total cholesterol	175 mg/dL	<200 mg/dL
Triglycerides	130 mg/dL	0-150 mg/dL
High-density lipoprotein cholesterol	49 mg/dL	>30 mg/dL
Low-density lipoprotein cholesterol	100 mg/dL	0-99 mg/dL
Very low-density lipoprotein cholesterol	26 mg/dL	10-30 mg/dL

Blood pressure became controlled, serum potassium normalized, direct renin became non-suppressed, and proteinuria improved on this regimen; therefore, telmisartan and cilnidipine were continued, and no further attempts were made to withdraw the renin-angiotensin system blockade. On follow-up, home blood pressure readings improved to approximately 130-140/80 mmHg. The patient also reported significant improvement in generalized and lower-limb weakness following correction of hypokalemia and initiation of mineralocorticoid receptor antagonist therapy. Given the presence of dyslipidemia and an elevated atherosclerotic cardiovascular disease (ASCVD) risk, rosuvastatin 10 mg was continued. The patient was counseled regarding lifestyle modifications, including weight reduction, dietary changes, and regular physical activity, in view of obesity and prediabetes.

## Discussion

In routine clinical practice, moderately elevated blood pressure in an older patient with obesity is frequently attributed to essential hypertension, and escalation of antihypertensive therapy may precede evaluation for secondary causes. The present case illustrates how such an approach can delay recognition of primary aldosteronism when warning signs, though subtle, are present. This patient’s hypertension began in early adulthood and was accompanied by persistent spontaneous hypokalemia with neuromuscular symptoms, proteinuria, and concentric left ventricular hypertrophy. The extent of renal and cardiac involvement appeared greater than expected for the degree of blood pressure elevation observed in clinic, and on home monitoring, consistent with evidence that PA is associated with disproportionate cardiovascular and renal injury compared with essential hypertension [2,3].

The aldosterone-renin ratio remains the cornerstone of screening for PA, but its interpretation is influenced by commonly used antihypertensive medications. The 2016 Endocrine Society guideline emphasized withdrawal of agents with major effects on renin and aldosterone when feasible and substitution with medications that minimally interfere with ARR interpretation [1]. However, the guideline also acknowledged that medication withdrawal may not always be safe or practical. The updated 2025 guideline further refines this approach by explicitly endorsing both minimal-withdrawal and no-withdrawal screening strategies, emphasizing contextual interpretation of ARR values rather than rigid medication cessation [4].

Beta-blockers and angiotensin receptor blockers (ARBs) exert opposing effects on ARR interpretation. Beta-blockers suppress renin release and may produce falsely elevated ARR values, whereas angiotensin receptor blockers increase renin and can lower ARR, potentially yielding false-negative results. When both drug classes are continued simultaneously, ARR becomes less reliable in isolation, as the opposing pharmacologic effects may obscure the true renin-aldosterone relationship. In this patient, angiotensin receptor blockade was initiated early because of clinically significant proteinuria, while beta-blocker therapy was gradually tapered and discontinued. This strategy was chosen to avoid simultaneous confounding effects on ARR while prioritizing renal protection and clinical safety. Correction of hypokalemia before testing was also essential, as low potassium can suppress aldosterone secretion and reduce diagnostic sensitivity [1].

Despite ongoing angiotensin receptor blockade, the patient demonstrated suppressed renin with inappropriately elevated aldosterone and a markedly elevated ARR, strongly supporting autonomous aldosterone secretion rather than a medication-related artifact. This aligns with current recommendations that emphasize absolute renin suppression and aldosterone excess, rather than ARR alone, when screening is performed on interfering medications [4].

Proteinuria in this case was clinically relevant rather than incidental, given its persistence and improvement following targeted therapy. Experimental studies demonstrate that aldosterone directly injures glomerular structures, including podocytes, through oxidative stress-mediated pathways, providing a mechanistic basis for proteinuria in aldosterone excess [6]. Clinically, patients with primary aldosteronism experience worse long-term renal outcomes than those with essential hypertension, even at comparable blood pressure levels [3]. In our patient, proteinuria improved after initiation of angiotensin receptor blockade and remained controlled following mineralocorticoid receptor antagonist therapy. This supports continuation of renin-angiotensin system blockade alongside spironolactone when blood pressure, potassium, renin levels, and proteinuria respond favorably, consistent with reports demonstrating that ARB therapy can mitigate proteinuria in medically treated primary aldosteronism [7].

The patient declined surgical intervention; therefore, medical therapy was pursued. Observational studies indicate that PA-specific medical treatment with MRAs is associated with improved cardiometabolic outcomes compared with nonspecific antihypertensive therapy, although residual risk may persist if aldosterone blockade is inadequate [8]. A large meta-analysis has further shown that PA confers a higher burden of cardiovascular events and target-organ damage than essential hypertension, underscoring the importance of disease-specific therapy rather than treating PA as conventional hypertension [9]. In this patient, spironolactone titration resulted in improved blood pressure control, normalization of potassium, and loss of renin suppression on follow-up, suggesting effective aldosterone blockade and appropriate dosing.

## Conclusions

This case underscores primary aldosteronism that should be suspected even in patients whose blood pressure elevation is modest relative to the degree of target organ involvement. Diagnostic strategies should be individualized and guided by clinical priorities; in this patient, angiotensin receptor blockade was initiated for renal indications, and aldosterone-renin testing was subsequently interpreted in the context of ongoing therapy. Such an approach facilitates timely recognition of aldosterone excess and highlights the importance of considering primary aldosteronism in the evaluation of patients with hypertension, with the potential to prevent progression to resistant hypertension and long-term cardiovascular and renal complications.
